# Supplementary material for: Uptake of COVID-19 Vaccines among Pregnant Women: A Systematic Review and Meta-Analysis
Source: Vaccines (Basel). 2022 May 12;10(5):766. doi: 10.3390/vaccines10050766 (PMC9145279; doi:10.3390/vaccines10050766)
Supplement: Supplementary file 1 [file vaccines-10-00766-s001.zip › Supplement Table S1.pdf]

Supplement Table S1a. Quality of cohort studies included in this systematic review.

	Rottenstreich et al. [1]	Blakeway et al. [2]	Wainstock et al. [3]	Razzaghi et al. [4]	Lipkind et al. [5]	Stock et al. [6]	UK Health Security Agency [7]	Goldshtein et al. [8]
1. Were the two groups similar and recruited from the same population?	Yes	Yes	Yes	Yes	Yes	Yes	Yes	Yes
2. Were the exposures measured similarly to assign people to both exposed and unexposed groups?	NA	Yes	NA	Yes	NA	NA	Yes	NA
3. Was the exposure measured in a valid and reliable way?	NA	Yes	NA	Yes	NA	NA	Yes	NA
4. Were confounding factors identified?	NA	Yes	NA	No	NA	NA	No	NA
5. Were strategies to deal with confounding factors stated?	NA	Yes	NA	No	NA	NA	No	NA
6. Were the groups/participants free of the outcome at the start of the study (or at the moment of exposure)?	Yes	Yes	Yes	Yes	Yes	Yes	Yes	Yes
7. Were the outcomes measured in a valid and reliable way?	Yes	Yes	Yes	Yes	Yes	Yes	Yes	Yes
8. Was the follow up time reported and sufficient to be long enough for outcomes to occur?	Yes	Yes	Yes	Yes	Yes	Yes	Yes	Yes
9. Was follow up complete, and if not, were the reasons to loss	Yes	Yes	Yes	Yes	Yes	Yes	Yes	Yes

to follow up described and explored?								
10. Were strategies to address incomplete follow up utilized?	No	No	No	No	No	No	No	No
11. Was appropriate statistical analysis used?	Yes	Yes	Yes	Yes	Yes	Yes	Yes	Yes
**Risk of bias**	**Moderate**	**Low**	**Moderate**	**Low**	**Moderate**	**Moderate**	**Low**	**Moderate**

NA: not applicable

Supplement Table S1b. Quality of cross-sectional studies included in this systematic review.

	Hosokawa et al. [9]	Taubman et al. [10]	Siegel et al. [11]
1. Were the criteria for inclusion in the sample clearly defined?	Yes	Yes	Yes
2. Were the study subjects and the setting described in detail?	Yes	Yes	Yes
3. Was the exposure measured in a valid and reliable way?	Yes	NA	Yes
4. Were objective, standard criteria used for measurement of the condition?	Yes	Yes	Yes
5. Were confounding factors identified?	Yes	NA	Yes
6. Were strategies to deal with confounding factors stated?	Yes	NA	Yes
7. Were the outcomes measured in a valid and reliable way?	Yes	Yes	Yes
8. Was appropriate statistical analysis used?	Yes	Yes	Yes
**Risk of bias**	**Low**	**Moderate**	**Low**

NA: not applicable

References

1. Rottenstreich, M.; Sela, H.; Rotem, R.; Kadish, E.; Wiener-Well, Y.; Grisaru-Granovsky, S. Covid-19 Vaccination during the Third Trimester of Pregnancy: Rate of Vaccination and Maternal and Neonatal Outcomes, a Multicentre Retrospective Cohort Study. *BJOG* **2022**, *129*, 248–255, doi:10.1111/1471-0528.16941.
2. Blakeway, H.; Prasad, S.; Kalafat, E.; Heath, P.T.; Ladhani, S.N.; Le Doare, K.; Magee, L.A.; O'Brien, P.; Rezvani, A.; von Dadelszen, P.; et al. COVID-19 Vaccination during Pregnancy: Coverage and Safety. *American Journal of Obstetrics and Gynecology* **2022**, *226*, 236.e1-236.e14, doi:10.1016/j.ajog.2021.08.007.
3. Wainstock, T.; Yoles, I.; Sergienko, R.; Sheiner, E. Prenatal Maternal COVID-19 Vaccination and Pregnancy Outcomes. *Vaccine* **2021**, *39*, 6037–6040, doi:10.1016/j.vaccine.2021.09.012.
4. Razzaghi, H.; Meghani, M.; Pingali, C.; Crane, B.; Naleway, A.; Weintraub, E.; Kenigsberg, T.A.; Lamias, M.J.; Irving, S.A.; Kauffman, T.L.; et al. COVID-19 Vaccination Coverage Among Pregnant Women During Pregnancy — Eight Integrated Health Care Organizations, United States, December 14, 2020–May 8, 2021. *MMWR Morb. Mortal. Wkly. Rep.* **2021**, *70*, 895–899, doi:10.15585/mmwr.mm7024e2.
5. Lipkind, H.S.; Vazquez-Benitez, G.; DeSilva, M.; Vesco, K.K.; Ackerman-Banks, C.; Zhu, J.; Boyce, T.G.; Daley, M.F.; Fuller, C.C.; Getahun, D.; et al. Receipt of COVID-19 Vaccine During Pregnancy and Preterm or Small-for-Gestational-Age at Birth — Eight Integrated Health Care Organizations, United States, December 15, 2020–July 22, 2021. *MMWR Morb. Mortal. Wkly. Rep.* **2022**, *71*, 26–30, doi:10.15585/mmwr.mm7101e1.
6. Stock, S.J.; Carruthers, J.; Calvert, C.; Denny, C.; Donaghy, J.; Goulding, A.; Hopcroft, L.E.M.; Hopkins, L.; McLaughlin, T.; Pan, J.; et al. SARS-CoV-2 Infection and COVID-19 Vaccination Rates in Pregnant Women in Scotland. *Nat Med* **2022**, *28*, 504–512, doi:10.1038/s41591-021-01666-2.
7. UK Health Security Agency *COVID-19 Vaccine Surveillance Report Week 47*; 2021;
8. Goldshtein, I.; Steinberg, D.M.; Kuint, J.; Chodick, G.; Segal, Y.; Shapiro Ben David, S.; Ben-Tov, A. Association of BNT162b2 COVID-19 Vaccination During Pregnancy With Neonatal and Early Infant Outcomes. *JAMA Pediatr* **2022**, doi:10.1001/jamapediatrics.2022.0001.
9. Hosokawa, Y.; Okawa, S.; Hori, A.; Morisaki, N.; Takahashi, Y.; Fujiwara, T.; Nakayama, S.F.; Hamada, H.; Satoh, T.; Tabuchi, T. The Prevalence of COVID-19 Vaccination and Vaccine Hesitancy in Pregnant Women: An Internet-Based Cross-Sectional Study in Japan. *Journal of Epidemiology* **2022**, JE20210458, doi:10.2188/jea.JE20210458.
10. Taubman – Ben-Ari, O.; Weiss, E.; Abu-Sharkia, S.; Khalaf, E. A Comparison of COVID-19 Vaccination Status among Pregnant Israeli Jewish and Arab Women and Psychological Distress among the Arab Women. *Nursing & Health Sciences* **2022**, nhs.12929, doi:10.1111/nhs.12929.
11. Siegel, M.R.; Lumbreras-Marquez, M.I.; James, K.; McBay, B.R.; Gray, K.J.; Schantz-Dunn, J.; Diouf, K.; Goldfarb, I.T. *Perceptions and Attitudes Towards COVID-19 Vaccination Amongst Pregnant and Postpartum Individuals*; Obstetrics and Gynecology, 2021;
