# Supplementary figures and images for: Uptake of COVID-19 Vaccines among Pregnant Women: A Systematic Review and Meta-Analysis
Source: Vaccines (Basel). 2022 May 12;10(5):766. doi: 10.3390/vaccines10050766 (PMC9145279; doi:10.3390/vaccines10050766)

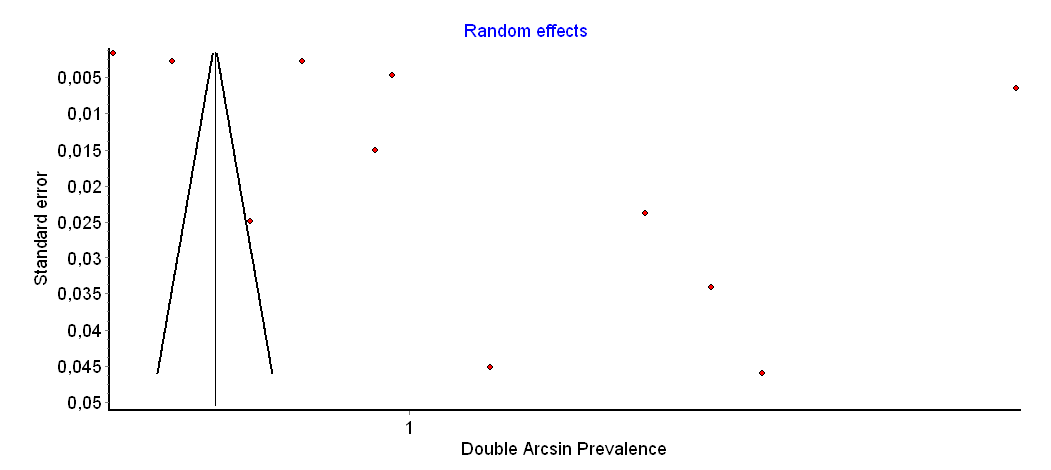

Supplement: Supplementary file 1 [file vaccines-10-00766-s001.zip › Supplementary Figure S1.png]
